# Experimental and Numerical Study on Impact Behavior of Hourglass Lattice Sandwich Structures with Gradients

**DOI:** 10.3390/ma16186275

**Published:** 2023-09-19

**Authors:** Hexiang Wu, Jia Qu, Linzhi Wu

**Affiliations:** 1 Key Laboratory of Advanced Ship Materials and Mechanics, College of Aerospace and Civil Engineering, Harbin Engineering University, Harbin 150001, China; 2 School of Civil Engineering and Transportation, Northeast Forestry University, Harbin 150040, China

**Keywords:** hourglass lattice sandwich structures, impact behavior, gradient, experiment, simulation

## Abstract

The impact mechanical properties of graded hourglass lattice sandwich structures under impact compression were studied using experiments and numerical simulations. The influence of the gradient distribution on the deformation mode, peak load, and energy absorption capacity of the hourglass lattice sandwich structure under the same impact energy level, different impact masses, and different impact velocities is discussed. The results show that the difference in impact mass and velocity has a significant effect on the impact mechanical properties of the graded hourglass lattice sandwich structure under the same impact energy level. The gradient distribution mode is a factor that requires careful consideration in the design. A reasonable gradient distribution design can control the initial and compression peak loads to achieve similarly low values and improve the load consistency of the hourglass lattice sandwich structure. The total energy absorption of the hourglass lattice sandwich structures with different gradient distributions is the same; however, the energy absorption capacity is different at different deformation stages. When the moving distance is 0.005 m, the gradient hourglass lattice sandwich structures with the mass decline distribution can absorb 1 kJ/kg more energy than the gradient hourglass lattice sandwich structures with the mass increment distribution. When the moving distance is 0.037 m, the mass decline distribution gradient hourglass lattice sandwich structures absorb 1 kJ/kg less energy than the mass increment distribution gradient hourglass lattice sandwich structures.

## 1. Introduction

The sandwich structure is lightweight, has high strength, high stiffness, and good energy absorption performance, and has functional characteristics such as sound insulation, noise reduction, thermal insulation, and shock buffering. It is widely used in military and civilian fields, such as aerospace, marine engineering, and construction engineering, for skin, cowling, propeller, and super high-rise building applications, and has attracted increasing attention from scholars and engineers [1,2,3,4,5]. Owing to the development in requirements for energy and material savings, the application range of sandwich structures has been further expanded, which has promoted comprehensive related research. A sandwich structure consists of an upper panel, lower panel, and welded or bonded middle core. The panel is mainly subjected to tensile and compressive stresses during impact, and is usually fabricated using high-strength metal. The core is mainly subjected to shear stress during impact, and is typically fabricated using foam, honeycomb, and lattice materials with light weight and high energy absorption [6,7,8].

According to the type of core, sandwich structures can be divided into foam, honeycomb, and lattice sandwich structures [9,10,11]. Foam and honeycomb sandwich structures are closed in the internal space and cannot achieve multifunctional characteristics such as embedment and heat transfer; thus, Hutchinson, Ashby, and Gibson et al. proposed lattice sandwich structures in the early 21st century to address this issue [12,13]. Lattice materials have quickly attracted the attention of researchers, and various topological configurations, such as tetrahedrons, 3D-kagome, and octahedrons, have been successively developed [14,15,16,17]. The preparation technology has a significant effect on the mechanical properties of lattice materials. Based on the existing configuration design of lattice materials, extrusion wire cutting, investment casting, additive manufacturing, hot pressing, one-time forming, interlock assembly, and interweaving processes have been developed, improved, and combined to obtain high-performance lattice materials [18,19].

In addition to engineering applications, research on lattice sandwich structures has focused on their static and dynamic properties. The study of static properties includes load-bearing capacity, failure mode, and energy absorption capacity. The results show that the load-bearing capacity, failure mode, and anti-buckling ability of the lattice sandwich structure are all affected to varying degrees by parameters and factors, such as the base material properties of the panel and core, thickness of the panel and core, density of the core, slenderness ratio of the rod, structural constraint form, and manufacturing defects [20,21,22]. The research of dynamic performance includes the influence of two conditions on the mechanical parameters and deformation characteristics, failure mode, ballistic limit velocity, and energy absorption capacity of the lattice sandwich structure. Design parameters of the sandwich structure, such as the panel material, panel thickness, density of the lattice material, and microtopology design of the lattice material, should be considered [23,24]. In addition, the differences in the impact characteristics, such as impact energy, location, and velocity, should also be considered [25]. Under an impact load, the mechanical behavior of materials will exhibit the characteristics of the inertia effect, strain rate effect, etc. The same material exhibits different dynamic characteristics under different impact velocities, energy levels, and impactor shapes [26,27,28,29].

Based on the concept of a functional gradient, a gradient sandwich structure with a cell configuration changing in a certain direction has been established. Early design and research on gradient sandwich structures mainly focused on gradient foam sandwich structures and gradient honeycomb sandwich structures [30,31]. The foam is a disordered core, and the gradient foam sandwich structure cannot be constructed by changing the geometric parameters of the cell. However, honeycombs are an ordered core, and a gradient honeycomb sandwich structure can be designed by changing its size, shape, and wall thickness. The static and dynamic mechanical properties of the gradient sandwich structure, such as compression, impact, and explosion resistance, have been studied using experimental and numerical simulation methods [32,33,34,35,36,37]. The results show that the gradient design can reasonably regulate the mass distribution in the honeycomb core, improve the specific strength and stiffness of the sandwich structure, and improve the bearing capacity of the structure under non-uniform loads. The design and research on gradient lattice sandwich structures began relatively late. Xu et al. designed and prepared an axial-gradient lattice sandwich structure by changing the span of the cells and width of the rods in 2015 [38,39]. The results showed that the mechanical properties per unit mass of the gradient lattice sandwich structure were better than those of the uniform lattice sandwich structure when the geometric parameters of the lattice material were distributed according to a certain gradient.

The magnitude of the impact energy level can be determined by controlling the impact mass and velocity. Currently, studies on the influence of various impact energy levels are mainly carried out by changing the impact velocity. Studies on the influence of the same impact energy level with different impact masses and velocities have not been reported. In addition, studies on the influence of gradient distribution on the dynamic performance of lattice sandwich structures are limited. Therefore, by incorporating experimental observation and numerical simulation methods, samples of a uniform hourglass lattice sandwich structure were prepared, a uniform hourglass lattice sandwich structure model and a gradient hourglass lattice sandwich structure model were established, and the effects of gradient design, impact mass, and impact velocity on the dynamic performance of the hourglass lattice sandwich structure were investigated and discussed. The aims of this study were to reveal the influence mechanism of gradient design on improving the dynamic properties of lattice sandwich structures, and to improve the load consistency and the controllability of energy absorption of lattice sandwich structures at the same time. The importance of this study is that the conclusions have reference value for safety protection devices in engineering.

## 2. Experimental and Simulation Methods

### 2.1. Specimen Manufacturing of Regular Sandwich Structures

**Figure 1:**
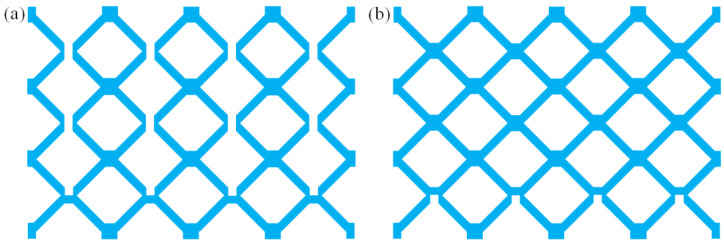
Interlocking parts of hourglass truss lattice: (**a**) part 1; (**b**) part 2.

**Figure 2:**
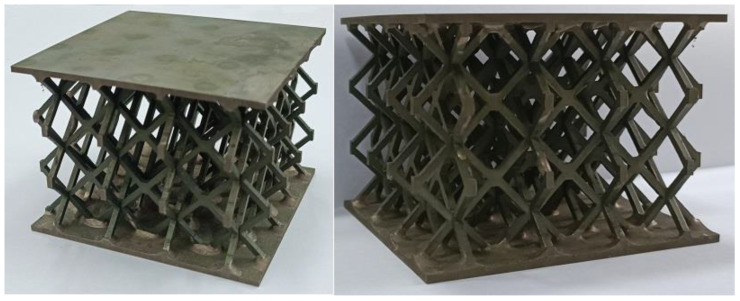
Specimens of the brazed hourglass lattice sandwich structure.

**Figure 3:**
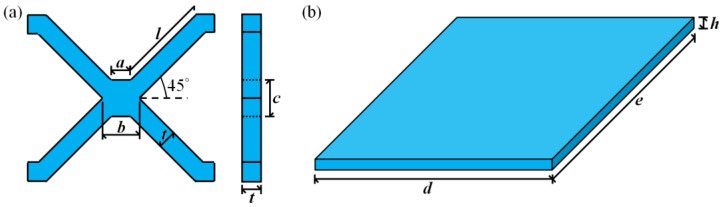
Geometries of the specimens: (**a**) interlocking parts of the representative unit cell of hourglass truss lattice; (**b**) panels.

Among the common lattice structures, hourglass lattice structures have a large elongated rod ratio, small node spacing, and strong antibuckling ability [40,41]. Therefore, this study investigates the impact response performance of an hourglass lattice sandwich structure. In this study, cutting–locking–brazing technology was used to prepare a multilayer hourglass lattice sandwich plate. A 304 stainless steel sheet was cut into parts 1 and 2 using electrical discharge machining wire-cutting technology, as shown in Figure 1. Components 1 and 2 were assembled into 3-layer hourglass lattice cores via locking, and the joints of the two locking components were vacuum-brazed to form an integral. The hourglass lattice core and upper and lower panels were also welded using vacuum-brazing technology to form an integral sandwich structure. In vacuum-brazing technology welding, the Nicrobraze 31 brazing solder was first placed at the node of the core, then the core was placed between the upper and lower panels, a load was lightly applied to ensure full contact between the panel and the core, and the overall sandwich structure was placed in a high-temperature vacuum-brazing furnace. It was heated to 950 °C at a rate of 15 °C /min, maintained at this state for 30–60 min, then heated to 1050 °C at a rate of 20 °C/min, maintained at a pressure of 2 × 10^−2^ Pa for 6–10 min, and finally cooled naturally to room temperature. The specimen of the prepared hourglass lattice sandwich structure is shown in Figure 2. The sandwich structure specimen has three layers of hourglass lattice structure, and each layer of the hourglass lattice structure has 4 × 4 hourglass lattice cells, each of which is composed of two interlocking components with the same size. The geometric sizes are shown in the front and side views in Figure 3a, where *a* = 2.35 mm, *b* = 3.85 mm, *c* = 3.85 mm, *l* = 9.7 mm, and *t* = 2 mm. The geometric dimensions of the square panel are shown in the stereogram in Figure 3b, where *d* = 79.672 mm, *e* = 79.672 mm, and *h* = 2 mm.

The relative density (Δ*ρ*) of the lattice structure is defined as the ratio of the lattice structure density (*ρ*_H_) to the base material density (*ρ*), as

(1) $$\Delta \rho =\rho_{H}/\rho ,$$

where the relative density of the hourglass lattice structure established in this study can be determined according to the proportion of the solid volume in the cell volume, that is

(2) $$\Delta \rho =\frac{t\left[8lt+2c\left(a+b\right)-ct\right]}{{\left(a+b+2l\cos45^{{}^{\circ}}\right)}^{2}\left(c+2l\sin45^{{}^{\circ}}\right)},$$

The relative density of the 3-layer hourglass lattice structure specimen prepared in this study is 0.056.

### 2.2. Drop Hammer Test

**Figure 4:**
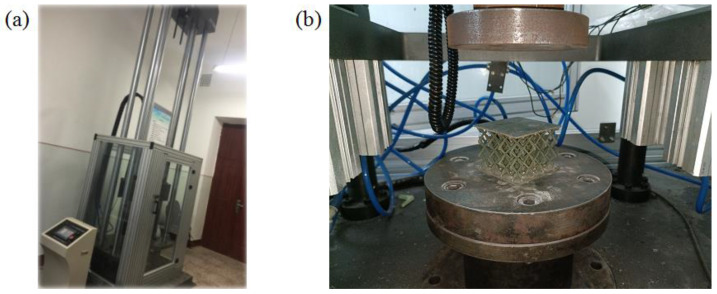
Drop hammer impact test: (**a**) JLY-6500 testing machines; (**b**) specimen and device.

Sandwich structures in engineering applications are inevitably impacted by external objects such as hail, birds, and floes. The drop-weight test is a simple test used to evaluate the impact resistance of materials [42]. During the test, the drop hammer is lifted to a certain height and released, and its potential energy is converted into kinetic energy and applied to the specimen to be tested. This study uses a JLY-6500 fully digital instrument-driven drop-hammer impact testing machine produced by KeXin in Changchun of China, as shown in Figure 4a. The column type sensor is used to measure the force. The maximum mass of the weight used by the testing machine is 240 kg, and the minimum mass is 100 kg. The hammer head is flat-bottom cylindrical, made of 42CrMo high-strength alloy steel, and quenched and tempered at a low temperature. The specimens were placed on a fixed base, as shown in Figure 4b. During the test, the change in the impact force history was measured using a load-measuring element above the hammer head. The experiments were repeated thrice to ensure the effectiveness of the results.

### 2.3. Finite Element Models of Graded Sandwich Structures

**Figure 5:**
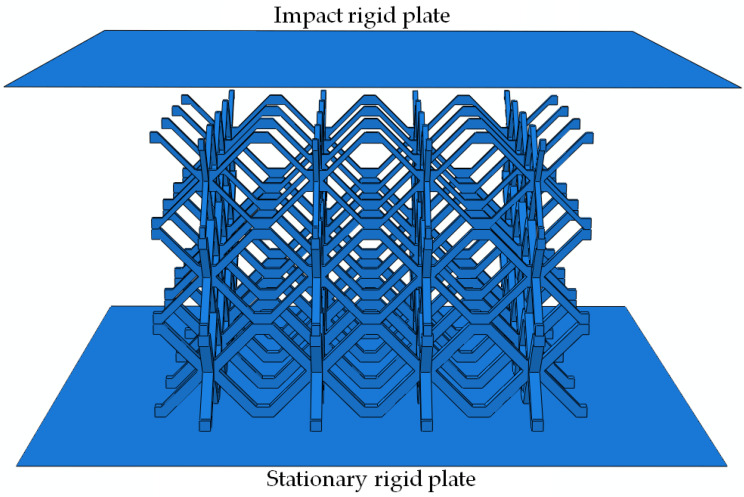
Calculating model of hourglass lattice structures.

The experimental cost of studying the impact performance of the gradient lattice sandwich structure is relatively high, and the mass and drop height ranges of the weight limit the adjustment range of the impact mass and velocity. Therefore, the numerical simulation method was used to further study the influence of different impact masses and velocities on the impact behavior of the graded hourglass lattice sandwich structure under the same impact energy level. The calculation model of the gradient hourglass lattice sandwich structure is shown in Figure 5. Considering that the upper and lower plates have little influence on the performance of the sandwich structure under flat plate impact compression, to save calculation costs, the calculation model only included three parts: the gradient hourglass lattice core, upper rigid plate, and lower rigid plate. This method has been considered in some studies on lattice structures [43,44]. The graded hourglass lattice core consists of three-layer uniform hourglass lattice structures with different relative densities. The relative density of the uniform hourglass lattice structure layer was changed by changing the thickness of the rod in the lattice structure. All the touching parts in the gradient hourglass lattice core were welded. During the analysis, the gradient hourglass lattice core was placed on a rigid plate with a fixed bottom end, and there were no constraints around it. The influence of the impact mass and velocity on the impact behavior of the graded hourglass lattice was studied by varying the mass and initial vertical velocity of the top rigid plate. Three groups were designed using the same impact energy level of 2000 J but different impact masses and velocities of 240 kg and 4.08 m/s, 18 kg and 14.91 m/s, and 2 kg and 44.72 m/s.

In this study, six gradient forms were considered: H_123_, H_132_, H_213_, H_231_, H_312_, and H_321_, where the subscript indicates the order of wall thickness from the impact end. The larger the number, the greater the thickness of the rod in the hourglass lattice structure layer. The relative density of the gradient hourglass lattice sandwich structure is

(3) $$\Delta \rho =\sum_{i=1}^{n}\frac{t_{i}\left[8lt_{i}+2c\left(a_{i}+b_{i}\right)-ct_{i}\right]}{{\left(a_{i}+b_{i}+2l\cos45^{{}^{\circ}}\right)}^{2}\left(c+2l\sin45^{{}^{\circ}}\right)},$$

where *n* is the total number of hourglass lattice structure cells, *t_i_* is the rod thickness of the *i*-th hourglass lattice structure cell, and *a_i_* and *b_i_* are geometric parameters corresponding to the *i*-th hourglass lattice structure cell.

Based on the calculation model shown in Figure 5, the impact behavior of the gradient hourglass lattice sandwich structure was studied using the ABAQUS 6.14. Through convergence analysis, the C3D10M explicit three-dimensional structural solid unit with a unit length of 1 mm was selected for the discrete model. In the compression process, surface-to-surface contact was defined between the rigid plate and the lattice structure, general contact was defined within the lattice structure, and the contact friction coefficient was 0.2 [45]. The base material of the sandwich structure was 304 stainless steel, and an ideal elastic–plastic model was adopted. The main material parameters were density *ρ* = 7800 kg/m^3^, Young’s modulus *E* = 213 GPa, Poisson’s ratio *ν* = 0.3, and yield strength *σ*_y_ = 212 MPa. Table 1 lists the geometric parameter values of gradient hourglass lattice structures with different arrangements, and provides the relative density values according to Equations (2) and (3), where *t*_1_, *t*_2_, and *t*_3_ represent the thicknesses of the rod in the first-, second-, and third-layer hourglass lattice structures of the hourglass lattice structure, starting from the top rigid plate to the bottom rigid plate.

## 3. Results and Discussion

### 3.1. Compressive Force of Regular Sandwich Structures

**Figure 6:**
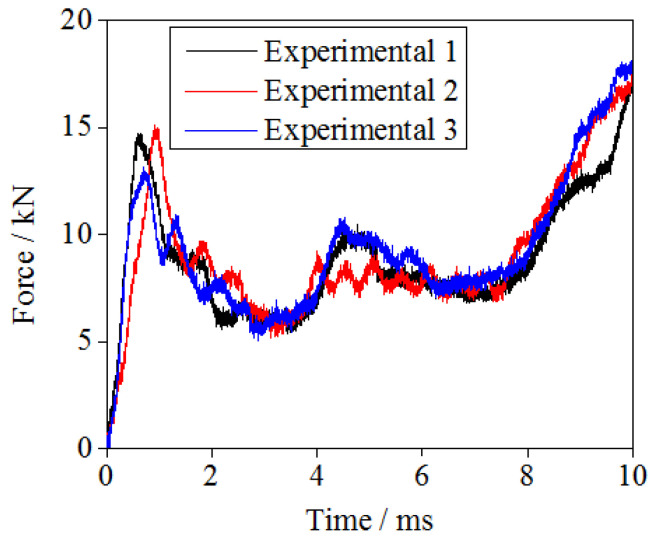
Experimental temporal compression force variations.

The regular hourglass lattice sandwich structures were tested using the drop hammer. The curve representing the relationship between compression load and time for the hourglass lattice sandwich structure under a 240 kg weight and 2000 J impact energy is shown in Figure 6. The results of the three repeated experiments are basically consistent; thus, they can be considered to be valid. In the impact compression process, the hourglass lattice sandwich structure first undergoes the elastic response stage, the core yields, and the compression load reaches a peak for the first time. As the loading continues, the core begins to soften, and the compression load curve exhibits a downward trend. This is because, when a plastic hinge is formed during the deformation process of the rod, the rod rotates around the plastic hinge, which increases the size of the force arm and reduces the force. Then, the compression load curve rises again, reaches a peak for the second time, and then gradually decreases. This is owing to the hardening of the base material in the deformation process, which improves the mechanical performance of the core; this is consistent with the results of some existing tests [46]. Finally, during the dense compression process of the core, the compressive load curve continues to rise until structural failure.

### 3.2. Validation of FE Models

**Figure 7:**
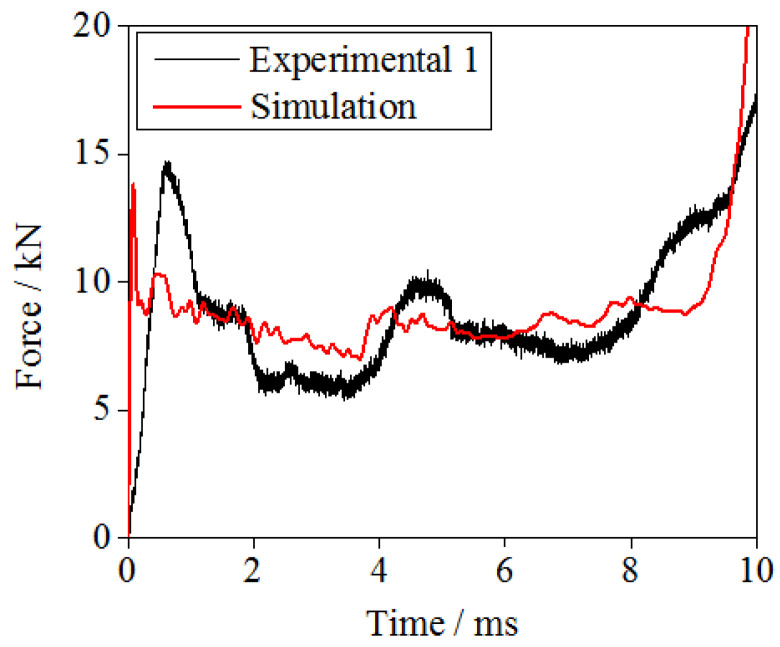
Comparison of load–time curve for hourglass lattice sandwich structures between the experiments and the simulations.

The dynamic performance of the gradient hourglass lattice sandwich structures are further studied by simulation. A finite element model with parameters the same as those in the experiment was established for verification purposes. The calculation model only considers the hourglass lattice core, and the settings of the boundary and loading conditions are exactly the same as the experimental conditions. Figure 7 shows the numerical simulation results of the load–time relationship curve of the hourglass lattice core during compression under the flat-plate impact condition and a comparison with the test results of the hourglass lattice sandwich structure. As shown in the figure, the numerical simulation results are in good agreement with the test results. Therefore, the numerical simulation method adopted in this study is reliable.

### 3.3. Deformation Mode of Graded Sandwich Structures

**Figure 8:**
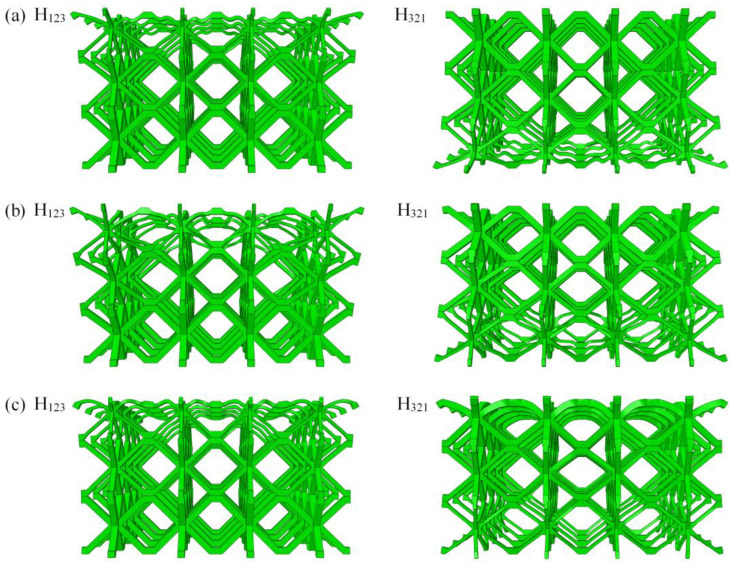
Damage morphology of gradient hourglass lattice structures at ε = 0.15: (**a**) 240 kg/4 m·s^−1^; (**b**) 18 kg/14 m·s^−1^; (**c**) 2 kg/44 m·s^−1^.

**Figure 9:**
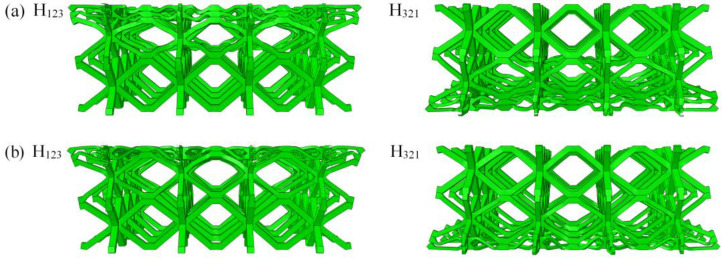

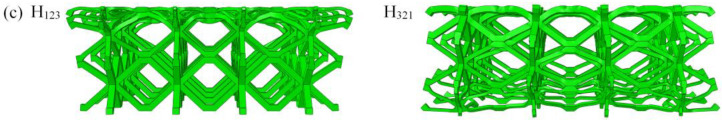
Damage morphology of gradient hourglass lattice structures at ε = 0.4: (**a**) 240 kg/4 m·s^−1^; (**b**) 18 kg/14 m·s^−1^; (**c**) 2 kg/44 m·s^−1^.

The deformation mode directly reflects the action mechanism in the impact compression process. In contrast to the solid panel structure, the lattice structure exhibits complex interactions during compression. Owing to space limitations, Figure 8 and Figure 9 only present deformation modes of H_123_ and H_321_ gradient hourglass lattice sandwich structures under relative compression processes with *ε* = 0.15 and *ε* = 0.4, under the same impact energy level, and different impact conditions of 240 kg/4 m·s^−1^, 18 kg/14 m·s^−1^, and 2 kg/44 m·s^−1^, where ε is the ratio of the distance moved by the impact rigid plate from contacting gradient hourglass lattice sandwich structure to the length of the gradient hourglass lattice sandwich structure along the impact compression direction. The results show that the deformation mode of the gradient hourglass lattice sandwich structure depends on the impact conditions.

As shown in Figure 8a and Figure 9a, under the condition of high-mass and low-velocity impact (240 kg/4 m·s^−1^), the deformation of the graded hourglass lattice sandwich structure starts from the low-density layer and causes layer-by-layer compression via local deformation until the high-density layer; for the H_123_ gradient hourglass lattice sandwich structure, the deformation starts from the impact end to the fixed end, whereas for the H_321_ gradient hourglass lattice sandwich structure, deformation starts from the fixed end to the impact end. The stress distribution quickly reaches equilibrium under low impact velocities, and the plastic collapse strength is the primary factor affecting the deformation mode. The plastic collapse strength of the low-density layer is low, whereas that of the high-density layer is high. With decreasing mass and increasing impact velocity (18 kg/14 m·s^−1^), the influence of impact velocity on deformation mode becomes apparent, as shown in Figure 8b and Figure 9b, respectively. The low-density layer and the structural layer at the impact end first deform in the initial stage, and then the deformation is concentrated in the low-density layer. The impact velocity is further increased and the mass is reduced (2 kg/44 m·s^−1^), as shown in Figure 8c and Figure 9c, where the impact velocity is the primary factor affecting the deformation mode. The deformation starts from the structural layer at the impact end and concentrates again in the low-density layer after the impact process. The length of the impact process is related to the impact velocity and dimensions of the gradient hourglass lattice sandwich structure along the impact direction.

### 3.4. Compressive Force of Graded Sandwich Structures

The impact compression load curve is the most accurate method for describing the mechanical response of a structure, where the load is the contact force between the hourglass lattice sandwich structure and impact rigid plate, and the displacement is the distance that the impact rigid plate moves from contact with the hourglass lattice sandwich structure. Based on the numerical simulation results of six types of graded hourglass lattice sandwich structures, the dynamic mechanical properties of the graded hourglass lattice sandwich structures under the same impact energy level, different impact mass, and velocity are discussed in this paper.

**Figure 10:**
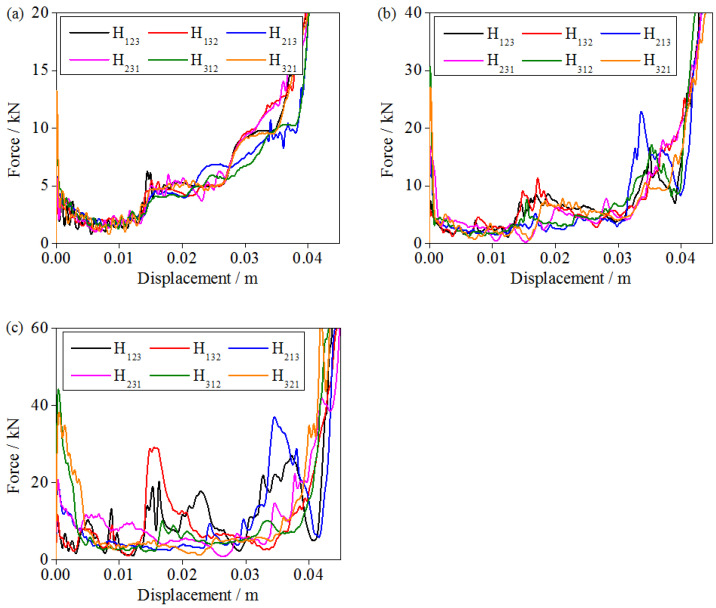
Impact force–displacement curves of various gradient hourglass lattice structures: (**a**) 240 kg/4 m·s^−1^; (**b**) 18 kg/14 m·s^−1^; (**c**) 2 kg/44 m·s^−1^.

As shown in Figure 10a, under the condition of high-mass and low-velocity impact (240 kg/4 m·s^−1^), the impact compression load increases from a low to a high value during the impact process after the initial peak load. This is because compression deformation occurs layer-by-layer from the low-density layer of the gradient hourglass lattice sandwich structure (the thickness of the rod is 1 mm) to the high-density layer (the thickness of the rod is 2 mm). The compression load of the graded hourglass lattice sandwich structure exhibits noticeable stage characteristics, and the response characteristics of the different graded hourglass lattice sandwich structures are very similar. Therefore, it can be concluded that, under the conditions of high-quality and low-velocity impact, the gradient form has little influence on the performance of the hourglass lattice sandwich structure and can be neglected.

With decreasing mass and increasing impact velocity (18 kg/14 m·s^−1^), the effect of gradient form becomes apparent. Figure 10b shows that, for the graded hourglass lattice sandwich structure with the same relative density, the stage characteristics of the compression load are noticeable, and the compression load increases over the duration of the impact process. However, the response characteristics of the hourglass lattice sandwich structures with different gradient forms began to show some differences. When the high-density layer is placed at the impact end, the compressive load of the gradient hourglass lattice sandwich structure in the early compression stage is higher. When the low-density layer is placed at the impact end, the compressive load of the gradient hourglass lattice sandwich structure in the early compression stage is lower. When the high-density layer is placed at the fixed end, the compression load of the gradient hourglass lattice sandwich structure is higher during the late compression stage. When the low-density layer is placed at the fixed end, the compression load of the gradient hourglass lattice sandwich structure is lower during the late compression stage.

As shown in Figure 10c, the gradient hourglass lattice sandwich structure exhibits a stronger dependence on the gradient form under the condition of low mass and high velocity impact (2 kg/44 m·s^−1^). The stress distribution in the model is significantly different among the hourglass lattice sandwich structures with different gradient distribution forms. In the impact compression process, the high and low orders of the phase compression load are the same as the numerical order in the subscript of the gradient distribution form. Taking H_312_ as an example, under an impact load, the phase order of the compression load is high (3), low (1), and medium (2).

### 3.5. Peak Force of Graded Sandwich Structures

**Figure 11:**
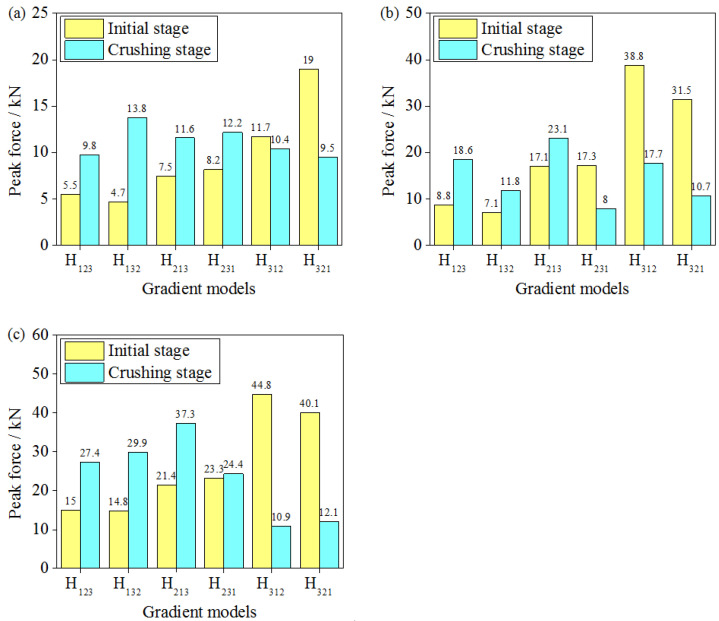
Peak force of various gradient hourglass lattice structures: (**a**) 240 kg/4 m·s^−1^; (**b**) 18 kg/14 m·s^−1^; (**c**) 2 kg/44 m·s^−1^.

A lightweight sandwich structure should maintain good load consistency during energy dissipation, and a variety of evaluation indices have been reported in relevant studies [47,48]. It is very important to control the peak force, which must be below the critical value to avoid direct damage to the energy-absorbing structure. The smaller the peak force, the better the load consistency of the lightweight sandwich structure and the more beneficial it is to the structure. Figure 11 shows the numerical simulation results for the peak force of the graded hourglass lattice sandwich structure under the same impact energy level, different impact masses, and different impact velocities, where the peak force includes the initial and compression stage peak forces. The initial stage peak force is the maximum load in the linear elastic deformation stage. The compression stage peak force is the maximum load after the linear elastic deformation stage and before the densification stage.

As shown in Figure 11, under the same impact energy level, different impact masses, and impact velocities, the peak force of the gradient hourglass lattice sandwich structure is different, showing an increasing trend with increasing impact velocity. The gradient form is an important factor that affects the peak force of the hourglass lattice sandwich structure, and the peak forces of the hourglass lattice sandwich structures with different arrangements are different. The order of the initial peak loads is the same as that of the number of density layers placed at the impact end. When a low-density layer is placed at the impact end, the initial peak load is low, whereas when a high-density layer is placed at the impact end, the initial peak load is high. Furthermore, when the low-density layer is placed at the impact end, the compression peak load exceeded the initial peak load, whereas when the high-density layer is placed at the impact end, the compression peak load is lower than the initial peak load. Lastly, when the initial and compression peak loads are similar and both low (such as the H_231_ arrangement), the gradient hourglass lattice sandwich structure can be considered to have excellent load consistency. From the above analysis, it can be observed that the impact velocity has a significant influence on the load consistency of the hourglass lattice sandwich structure, and the gradient arrangement is also an important influencing factor that cannot be ignored. Under different impact velocities, the load consistencies of the gradient hourglass lattice sandwich structures with different arrangement differ.

### 3.6. Energy Absorption of Graded Sandwich Structures

An important application of lightweight sandwich structures is to provide a buffer space through pores and absorb impact energy through structural deformation when subjected to impact loads. The energy absorption per unit mass is often used to characterize the energy absorption capacity of a sandwich structure, as [49]

(4) $$E_{m}=\frac{E_{v}}{\Delta \rho \rho },$$

where *E_v_* represents the strain energy per unit volume, that is,

(5) $$E_{v}=\int_{0}^{\varepsilon_{d}}\sigma d\varepsilon ,$$

where *σ* represents the nominal stress, defined as the ratio of the contact force between the impact rigid plate and specimen to the initial cross-sectional area of the specimen. *ε* represents the nominal strain. *ε_d_* represents the densification strain, defined as the strain at which the sandwich structure is completely compressed.

**Figure 12:**
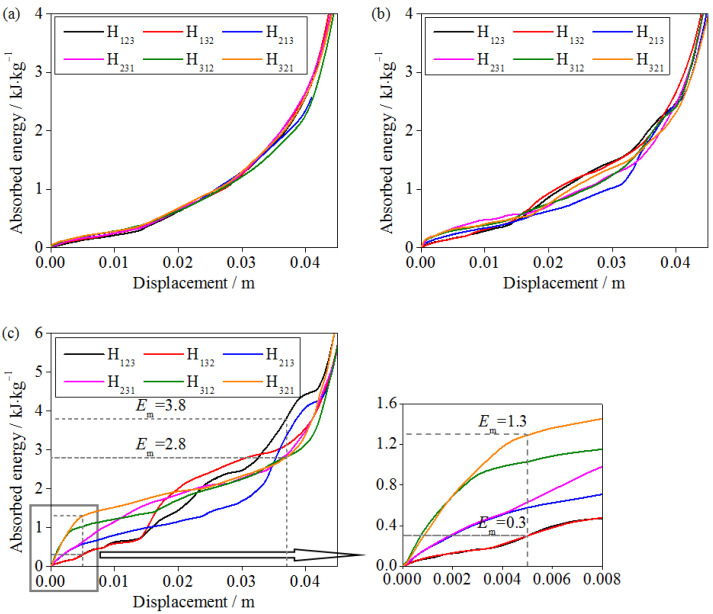
Energy absorption of various gradient hourglass lattice structures: (**a**) 240 kg/4 m·s^−1^; (**b**) 18 kg/14 m·s^−1^; (**c**) 2 kg/44 m·s^−1^.

Based on Equation (4), under the impact conditions of 240 kg/4 m·s^−1^, 18 kg/14 m·s^−1^, and 2 kg/44 m·s^−1^, the energy absorption per unit mass of the gradient hourglass lattice sandwich structure is shown in Figure 12. It ca n be seen from the figure that under the conditions of high-mass and low-velocity impact, the energy absorption processes of the hourglass lattice sandwich structures with different gradient forms are highly consistent, and the influence of the gradient forms can be negligible. As the mass decreases and the impact velocity increases, the role of the gradient form begins to appear. Under the conditions of low-mass and high-velocity impacts, the energy absorption depends more on the gradient distribution. In the impact compression process, the energy absorption capacities of different gradient hourglass lattice sandwich structures show relative changes. Taking the H_123_ and H_321_ hourglass lattice sandwich structures as examples, when the moving distance is 0.005 m, the gradient hourglass lattice sandwich structures with the H_321_ distribution can absorb 1 kJ/kg more energy than the gradient hourglass lattice sandwich structures with the H_123_ distribution. When the moving distance is 0.037 m, the H_321_ gradient hourglass lattice sandwich structures absorb 1 kJ/kg less energy than the H_123_ gradient hourglass lattice sandwich structures. When the impact compression structure reaches the densification stage, the total energy absorption of the hourglass lattice sandwich structures with different gradient forms is almost the same.

## 4. Conclusions

In this study, a gradient hourglass lattice sandwich structure finite element model was established by using ABAQUS 6.14, and the influence of the gradient distribution on the impact performance of the hourglass lattice sandwich structure under the same impact energy level, different impact masses, and different impact velocities is discussed using a simulation method. The reliability of the simulation method is proved by the good agreement between the numerical simulation results and the experiments results.

Under the conditions of high-mass and low-velocity impacts, the deformation starts from the low-density layer of the model to the high-density layer, and the impact load increases continuously with the impact process after the initial peak load. The density of the structural layer placed at the impact end affects the initial peak load level. Under the condition of low-mass and high-velocity impact, the response of the gradient hourglass lattice sandwich structure shows characteristics related to the gradient distribution, and the gradient distribution mode should be carefully considered in the design. Through a reasonable gradient distribution design, the initial peak load and compression peak load can be controlled to obtain similarly low values, and the load consistency of the hourglass lattice sandwich structure can be improved. Although the total energy absorption is the same, the energy absorption capacities of the hourglass lattice sandwich structures with different gradient distributions differ at different deformation stages.

In a word, the gradient arrangement can improve the safety of the structure under an impact load, control the impact mechanical properties of the structure, and provide a new design idea for safety protection structures in engineering.

## Figures and Tables

**Table 1:** Geometric parameters of gradient hourglass lattice sandwich structures with different gradient profiles.

Specimens	*t*_1_ (mm)	*t*_2_ (mm)	*t*_3_ (mm)	*l* (mm)	*a* + *b* (mm)	*c* (mm)	Δ*ρ*
H_123_	1	1.5	2	9.7	6.2	3.85	0.0358
H_132_	1	2	1.5				
H_213_	1.5	1	2				
H_231_	1.5	2	1				
H_312_	2	1	1.5				
H_321_	2	1.5	1				

## Data Availability

No new data were created or analyzed in this study. Data sharing is not applicable to this article.
